# Cytokinin and Its Key Role to Enrich the Plant Nutrients and Growth Under Adverse Conditions-An Update

**DOI:** 10.3389/fgene.2022.883924

**Published:** 2022-06-20

**Authors:** Ravindra Prasad

**Affiliations:** Department of Genetics and Plant Breeding, Institute of Agricultural Sciences, Banaras Hindu University, Varanasi, India

**Keywords:** wheat, spot blotch, nutrients, cytokinin (CK), CKX, biofortification, stress

## Abstract

Among the field crops, wheat is regarded as one of the most paramount cereal crops because it is widely grown, consumed as food across the world, and also known as the staple food for nearly 35 per cent of the world population. However, it is threatened by spot blotch disease causing considerable yield loss, with existing genotypes lacking the resistance and the necessary nutrients. Cytokinins (CKs) are key phytohormones that not only regulate the plant growth/development but also play an important role during stress and in the nutrient metabolic pathway of crop plants. Deficiency of important nutrients like zinc, iron, and vitamin A causes irreparable damage to the body, pressing the need to increase the accumulation of such micronutrients in the edible parts of the plant. Crop bio-fortification is one of the emerging approaches through which the quantities of these nutrients could be increased to an advisable amount. Cytokinin is observed to have a pivotal role in managing environmental stress/climate change and defense systems of plants, and apart from this, it is also found that it has an impact over Zn accumulation in cereal crops. Manipulation of the cytokine dehydrogenase (CKX) enzyme that degrades cytokinin could affect the yield, root growth, and important nutrients. Several instances revealed that an increment in the contents of Zn, S, Fe, and Mn in the seeds of cereals is a reflection of increasing the activity of CKX enzyme resulting the enhancement of the root system which not only helps in the absorption of water in a drought prone area but is also beneficial for scavenging nutrients to the deeper ends of the soil. Exploring micronutrients from the lithosphere *via* the root system helps in the uptake of the micronutrients and transporting them *via* the vascular system to the sink of crop plants, therefore, identification and incorporation of CKs/CKX linked gene(s) into targeted crop plants, exploring a bio-fortification approach including *CRISPR-Cas9* through conventional and molecular breeding approaches could be the most paramount job for improving the important traits and stress management in order to enhance the plant growth, productivity, and nutritional value of the wheat crops, which would be useful for mankind.

## Introduction

Wheat (*Triticum aestivum* L.), belongs to the family Poaceae*,* and is one of the most economically important cereal crops in the world (Schnurbusch, 2019; Alzaayid and Aloush, 2021). Among the field crops, wheat is regarded as the most crucial cereal crop because it is widely grown and consumed as food around the world. It is also known as the staple food for nearly 35 per cent of the world population and demand for wheat is expected to grow faster than the other major crops (Pingali, 2000). It is being cultivated in a wide range of environmental conditions across the globe and it is rich in nutrition components and provides approximately 20% of protein in the human diet (Reynolds and Braun, 2019). Much of the success was caused by the combination of high rates of investment in crop research, infrastructure, market development, and appropriate policy support that took place during the first Green Revolution, but still there is need to improve the crop productivity to meet the demand of the rapidly growing population (Pingali, 2000). Wheat, however, is being cultivated at a large scale and there is maximum demand in the world, so in response to food security in 21st century, structural transformations are needed to improve the crop yield in order to meet the demand of needy people (Pingali, 2015). Instead of just being considered a staple food, wheat is also a staple source of nutrients for around 40% of the world (Giraldo et al. (2019). However, malnutrition is still a serious issue these days, hence, the development of the promising wheat genotypes through crop bio-fortification which helps the conventional and advance breeding approach is required for nutritional security in the 21st century (Sujatha et al., 2021). Approximately 90%–95% of the wheat produced in the world is common or bread wheat having 2*n* = 6*x* = 42 composed of three sub genomes like A, B, and D diploid genomes which is a rich reservoir of genes determining yield and its contributing traits (Nadolska et al., 2017).

However, wheat crop is threatened by spot blotch disease caused by *Bipolaris sorokiniana* syn. *Helminthosporium sativum* syn. *Cochliobolus sativus* which is considered one of the most devastating diseases in Eastern India and South East Asia (Joshi et al., 2002); Kumar et al. (2009). Globally it is known as the most important disease, mainly in warm and humid regions of South Asia and South America (Kumar et al., 2019). As per challenges, efforts have been made and several resistance genotypes have been identified but availability of immune or near immune plants is lacking (Lillemo et al., 2012; Kumar et al., 2019). This disease was reported in the beginning of 19th century but gained more importance after the Green Revolution (Saari, 1998). Sensitive cultivars of barley and wheat are under severe attack from pathogens mainly at the time of late milking, dough stage, or at the time of flowering which badly disturbs the grain filling and eventually leads to lowering the yield of barley (Prasad et al., 2013) and wheat crops (Gupta et al., 2018). The application of fungicide can completely control/reduce the spot blotch disease severity (Videma and Kohli, 1998), but repeated application of such fungicides not only increases the cost of cultivation but also pollutes the environment, and is associated with emergence of fungicidal resistance in the target pathogen as well (Golembiewski et al., 1995). Hence, development of resistance cultivars by combining the conventional and advance molecular breeding approach is an effective and cost-effective strategy for combating the spot blotch problem. The availability of genetic information on spot blotch resistance genetics is scant as revealed by available literature and very limited genotypes have had their resistance level identified. Besides this disease, wheat crop also suffers from other biotic and abiotic factors as well as lacking the necessary nutrients for the human body. When the body does not get enough nutrients, it creates many problems including digestion, fatigue, dizziness, weight loss, and malnutrition which can cause physical or mental disability as well. Esra Koç and Belgizar Karayiğit (2022) stated that micronutrients are essential for physiological functions, and their deficiency causes serious health disorders, and it (Zn) is effective in many events such as reproduction and neurotransmission, especially the immune system. Similarly, Verma et al. (2021) reported that deficiency of micronutrients causes impairments to learning, physical growth, and reproductive health, decrease in immune resistance, and an increase in the rate of infection too.

Although the country has achieved total food grain production estimated at 296.65 million tonnes during 2019–20, this production is/was higher by 26.87 million tonnes than the average production of food grain of the previous 5 years. Similarly, wheat production is estimated at 107.59 million tones during 2019–20 (Anonymous). This bumper food grain production is primarily attributed to the production of high yielding genotypes. However, during the production of high yielding varieties, enough attention has not been given towards important nutrients; as a result, such improved genotypes are high yielding but have low concentrations of important nutrients as the standard recommended level. Thus, exploring the genetic information and identifying potential genotypes which confer a good source for resistance, are rich in important nutrients, and can harness the cytokinin hormone associated genes would be a wonderful approach for improving crop yield and other desirable traits using conventional and molecular breeding techniques including multidisciplinary approaches. In light of producing the high yielding wheat genotypes along with those rich in targeted nutrients and desirable for other agronomically traits, harnessing the cytokinin phyto hormones are one of the emerging approaches for the researchers under the changing climate. The changing climate results in an increase of the greenhouse gases which cause the reduction of crop production, yield, quality, and as result, nutritional deficiency in humans (Myers et al., 2014). Moreno-Jiménez et al. (2019) stated that increasing drought is because of changing the climatic and it lowers the availability of essential micronutrients, especially Fe and Zn. In order to supply the demand of the rapidly growing population, increasing the yield of crop plants is the prime objective of plant breeders/scientists in the 21st century as well as producing enough food, increasing the important nutrients level in the edible parts of crop plants, through either bio-fortification (producing crops that have higher levels of nutrition in their edible parts) or developing the superior genotypes along with rich in nutrients through modern breeding approaches is the current need. Since malnutrition is a challenging issue at a global level, efforts have been made under the leadership of the Consultative Group on International Agricultural Research (CGIAR) and accordingly huge bio-fortified genotypes of different crop plants have been developed across the globe. In continuation, in the country, 28 bio-fortified wheat genotypes along with those better for other desirable traits have been developed and released (Yadava et al., 2022). Yield of wheat or targeted crop plants including other desirable traits can also be enhanced by two key methods, the first is developing the high yielding genotypes as well as those better for resistance and rich in important nutrients by incorporating all linked gene(s)/QTLs into a single cultivar, and the second is saving the potential yield loss of genotypes occurred by either biotic or abiotic factors.

## Harnessing the Cytokinin and Its Role for Enhancing the Crop Yield and Other Traits

**FIGURE 1:**
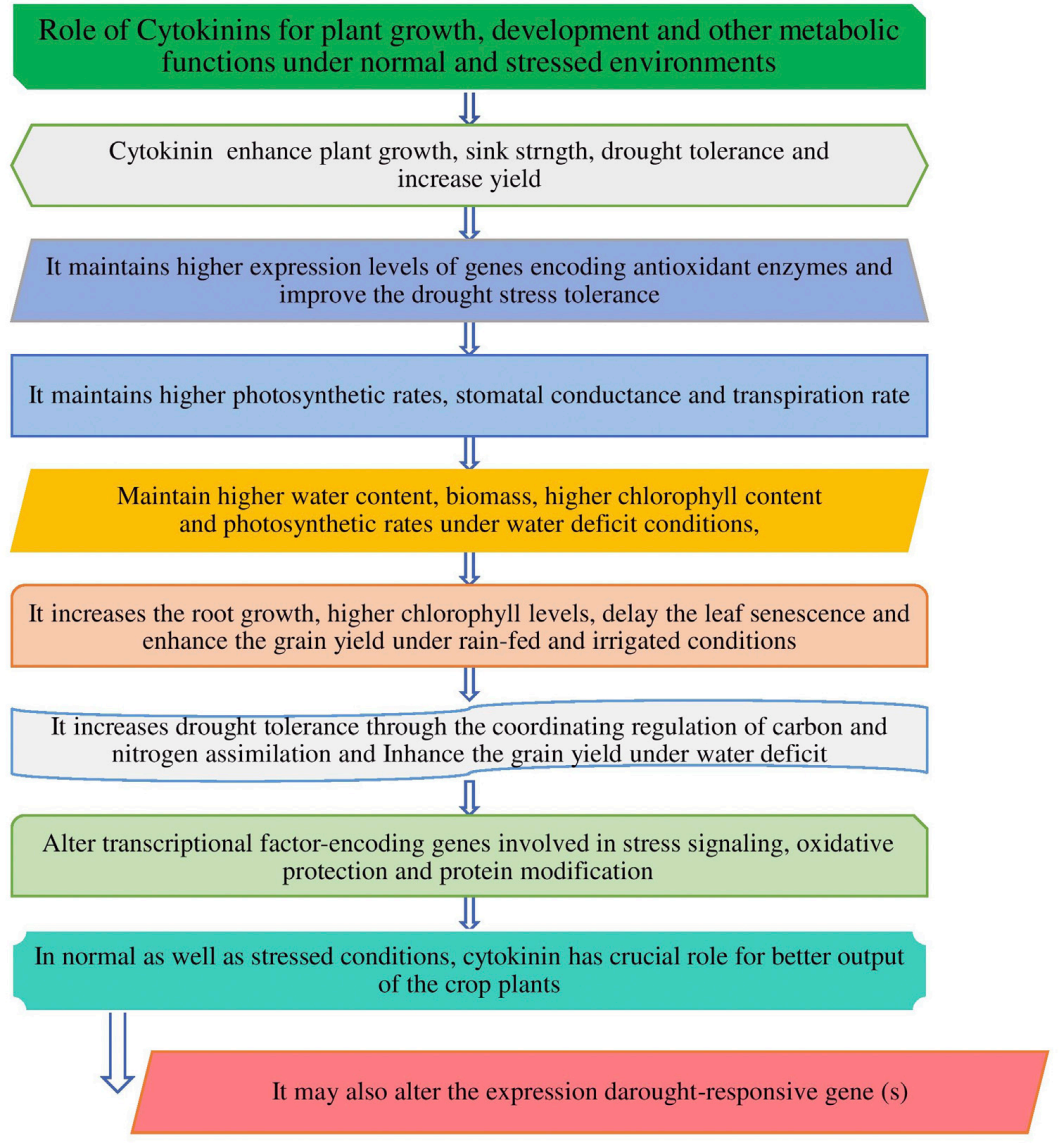
Cytokinin and its major role in crop plants in normal and stressed conditions.

**FIGURE 2:**
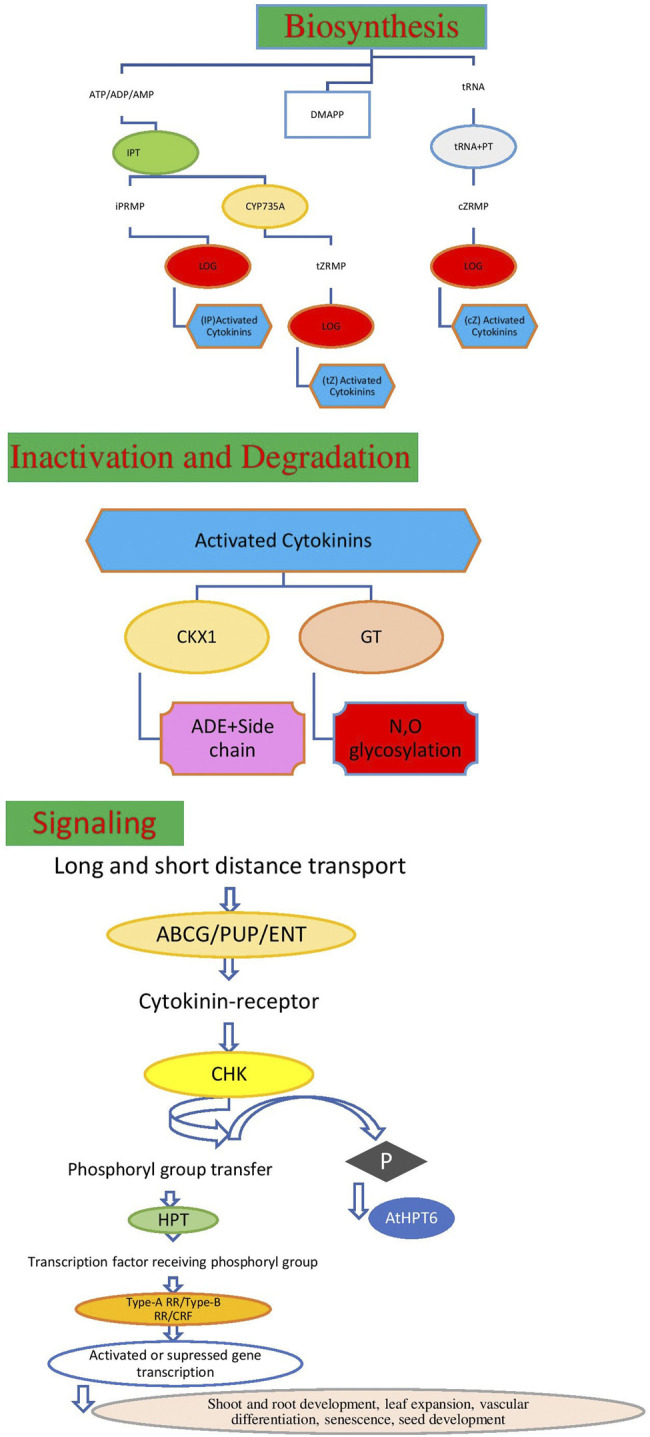
Model of cytokinin biosynthesis, metabolism, degradation and signaling dransduction process.

Hormones are produced naturally by plants, while plant growth regulators are applied to plants by humans for specific purpose. Plant growth hormones are essential components and control the overall outcome of plant growth and development (Mishra et al., 2022). Plant hormones and growth regulators are chemicals that affect the flowering, ageing, root growth, distortion and killing of organs, prevention or promotion of stem elongation, color enhancement of fruit, prevention of leafing, leaf fall, etc. There are five main groups of plant-growth-regulating compounds such as cytokinin, auxin, gibberellin (GA), ethylene, and abscisic acid (ABA) which are studied by the researchers, however, out of five groups, cytokinin plays a very crucial role for the cell cycle and affects the plant growth, development (Honig et al., 2018), promoting cell division and other physiological processes including germination, flowering, seed development, and leaf senescence, etc. as also presented in Figure 1. CKs have a significant impact on regulation of plant growth, stabilization of photosynthetic machinery during stress and exogenous application and modulation of CK levels have a positive effect on drought tolerance (Rivero et al., 2007). Research findings shows that cytokinins can alleviate the damage to plants caused by a variety of abiotic stresses (Mi et al., 2017; Prerostova et al., 2018). ABA is also considered the most important hormone as it controlled the plant water loss, hence it plays a key role under water-limited conditions for plant survival (de Ollas and Dodd, 2016. So, hormones are being exploited for crop improvement for traits of interest under normal as well stressed conditions (Gan and Amasino, 1996; Haberer and Kieber, 2002). Unlike other hormones, cytokinins are found in both plants and animals and they are considered to be master regulators of plant growth and development as well as being involved in the regulation of many important physiological and metabolic processes in crop plants (Wu et al., 2020) as it is also shown in Figures 1, 2, respectively. Chemically, natural cytokinins are N6-substituted purine derivatives. Isopentenyladenine (iP), zeatin (Z), and dihydrozeatin (DZ) are the predominant cytokinins found in higher plants. The free bases and their ribosides (iPR, ZR, DZR) are thought to be the biologically active compounds. Glycosidic conjugates play a role in cytokinin transport, protection from degradation, and reversible/irreversible inactivation. There are over 20 different forms of cytokinins that have been reported in wheat by Sykorova et al. (2008) and most of them are able to interconvert to release the active free bases.

Climate change is expected to continue posing either biotic or abiotic stresses globally, which leads to hampering the agricultural practices and limiting the yield of crop plants. The investments in exploring and harnessing the genes associated with desirable traits and beneficial roles of cytokinin phytoharmone for enhancing the plant production under stressed as well as efficient agricultural production systems are required to ensure the availability of enough food with nutrition security in the face of climate change. Stable or well adapted genotypes and the roles of cytokine hormones can thrive in challenging situations. Cytokinin plays diverse roles (Figures 1, 2) in response to plant growth and development, influencing many agriculturally important processes, including growth, nutrient responses, and the response to biotic and abiotic stresses (Liu et al., 2020), but under adversity stress, mechanisms of cytokinin-alleviating stress are different under different stresses, and its levels in plants are regulated by biosynthesis and inactivation pathways in crop plants.

In addition to genetic approaches for enhancing the impotent/essential nutrients, grain Zn concentration in wheat can also be increased by applying Zn-containing fertilizers, a process termed agronomic bio-fortification or agro-fortification (Zhao et al., 2019). In a review of published field studies, Joy et al. (2015b) noted that foliar application of Zn (ZnSO_4_) fertilizers, applied as a drench to field-grown wheat that can increase the whole-grain Zn concentration by a median of 63%. Soil-applied Zn fertilizers can also increase grain Zn concentrations, albeit to a much lesser extent than foliar-applied Zn fertilizers but may also increase crop yield (Zou et al., 2012). Similarly, Zia et al. (2020) carried out the field experiment and reported that *Zincol-2016*, a new variety of wheat, contains a higher concentration of Zn in its grain but other wheat genotypes responded to substantially increasing the grain Zn concentration when foliar Zn fertilizers are applied. However, application of soil Zn fertilizers had no significant effect on grain Zn concentration.

## Cytokinin Genes and their Association/Expression for Yield and Yield Related Traits in Wheat

Szala et al. (2020) studied the effect of *TaCKX* wheat gene family members (GFMs) encoding the enzyme cytokinin oxidase/dehydrogenase (CKX), which irreversibly degrades cytokinins (Werner et al., 2006), and therefore strongly regulates cytokinin contents in different parts of plants. Their findings revealed the presence of natural variation in expressive levels of tested genes in controlled and normal field conditions which was very high for yield and its contributing traits, indicating the possibility of selection of beneficial wheat genotypes for breeding and enhancing the yield of crops. Cytokinins also play a diverse role in plant development and affect a number of agriculturally important processes (Jameson and Song, 2016; Kieber and Schaller, 2018). In cereal crops, silencing of selected *CKX* is well documented in rice (Ashikari et al., 2005), in barley (Zalewski et al., 2010; Zalewski et al., 2012) and in wheat (Jablonski et al., 2020), leading to an increased level of cytokinins influencing the yield and its component traits. The number of *CKX* GFMs varies depending on species, however, in bread wheat 11 to 14 gene family members have been proposed (Ogonowska et al., 2019; Shoaib et al., 2019). Like wheat, in barley, Zalewski et al. (2014) studied the expression pattern of *HvCKX* and reported that these genes have a crucial role in the growth and reproductive development of barley crops. Similarly, Ogonowska et al. (2019) investigated the *CKX* genes in wheat with an aim to know the expression specificity of such genes for different developmental stages of crops of the plants and based on the expression of genes, they have classified such genes into four following groups:

1) Leaf-specific e.g., *TaCKX9*, *TaCKX5*, *TaCKX4*
2) Inflorescence specific and developing spike e.g., *TaCKX1* and *TaCKX2*
3) Seedling root-specific e.g., *TaCKX10*, *TaCKX7,* and
4) Expressed at various levels in all tested organs e.g., *TaCKX11*, *TaCKX3*, *TaCKX8.*

Jablonski et al. (2020) also studied the effect of *TaCKX1* silencing in wheat and reported that it was influenced by different modes of co-expression with other *TaCKX* GFMs and parameters of yield-related traits as well. Each of the tested yield-related traits was regulated by various up or downregulated *TaCKX* GFMs and phytohormones. According to Shanks et al. (2018), cytokinins regulate gene transcription in targeted organs and developmental stages of crops are associated with a wide range of transcription factors (TFs). One of the largest groups of plant TFs involved in cytokinin-dependent regulation is the family of NAC (for NAM, ATAF, and CUC) TFs. It has been documented that NACs are involved in the regulation of important agronomic traits. Alzaayid and Aloush (2021) conducted the field experiment with an aim to investigate the effect of spraying cytokinins of different concentrations on the growth and yield of wheat varieties and found that ten wheat cultivars showed a significant difference in growth, yield, and quality. Higher concentrations of cytokinins indicated a significant difference for the most of traits such as flag leaf area, number of grains, biological yield, and protein percentage respectively. Hence, identified genotypes may be used as a parent or donor under breeding programs for improving the yield and its attributing traits as well.

Cytokinins biosynthesis, metabolism, degradation, and signaling transduction processes of cytokinins are presented in Figure 2, which were already described by Wu et al. (2020). Gene(s) currently known to be involved in the cytokinin biosynthesis pathway and encode the *isopentenyl transferase* (IPT) and lonely guy (LOG) enzymes are reported by Takei et al., 2001 and Kurakawa et al., 2007. The initial step of cytokinin biosynthesis in higher plants is the formation of cytokinin nucleotides, namely, isopentenyladenosine 5′-tri-, di-, or monophosphate (iPRTP, iPRDP, or iPRMP, respectively from ATP, ADP, or AMP and dimethylallyl pyrophosphate (DMAPP) by IPTs5. LOGs, which encode phosphoribohydrolase-activating enzymes, directly convert a cytokinin nucleotide to an active free-base form of cytokinins in the final step of cytokinin biosynthesis while the levels of active cytokinins can be modulated *via* irreversible cleavage by cytokinin oxidase (*CKX*) enzymes (Schmulling et al., 2003) or through conjugation to glucose by cytokinin glycosyltransferases (Hou et al., 2004). Plants regulate the concentration of active cytokinins through either reversible or irreversible metabolism processes. Therefore, the precise maintenance of the homeostasis of cytokinins through these synthesis and inactivation enzymes is essential for plant development and adaptation. The full forms of the abbreviations used in Figure 2 are DMAPP: dimethylallyl pyrophosphate; iPRMP: isopentenyladenosine-5-monophosphate; tZRMP, trans-zeatin riboside 5′-monophosphate; cZRMP, cis-zeatin riboside 5′-monophosphate; iP, N6-(Δ2-isopentenyl) adenine; tZ: trans-zeatin; cZ: cis-zeatin; Ade: adenine; IPT, isopentenyltransferases; tRNA-IPT, tRNA-isopentenyl transferase; CYP735A, cytochrome P450 monooxygenase; LOG, LONELY GUY; GT, glycosyltransferase; CKX, cytokinin oxidase/dehydrogenase; ABCG, g subfamily ATP-binding cassette; PUP, purine permeases; ENT, equilibrative nucleoside transporters; HKs, histidine kinase; HPTs, histidine phosphotransfer proteins; ARR, response regulator, CRF, cytokinin response factor, etc. Cytokinin signal transduction pathway is also presented in Figure 2, and to date it has been well studied by researchers. In microorganisms a two-component system (TCS) is applicable, which changes the gene expression levels and acts in response to various stimuli and improve their ability to recognize and adapt to environmental changes (Cheung and Hendrickson, 2010). This TCS includes the flowing proteins: histidine kinases (HKs) associated with the membrane and response regulators (RRs) in the cytoplasm, HKs detect the environmental input in the sensor area and transmit the generated signal to the cytoplasm as reported by Cheung and Hendrickson (2010). However, based on TCS, plants have evolved a multi-step phosphorylation system, including the following three components: HKs, histidine phosphotransfer proteins (HPs), and RRs presented in Figure 2 and reported by Grefen and Harter (2004). Cytokinin uses this multi-step phosphorylation system for its signal transduction, including participation in cell division, leaf senescence, and apical dominance (Pils and Heyl, 2009). Mishra et al. (2022), stated that plant growth hormones are essential components that control the overall outcome of the growth and development of the plant, while cytokinins are hormones that play an important role in plant immunity and defense systems. In order to show this, they have identified nine functional modules comprised of different hub genes 36) which contribute to the cytokinin signaling route. Out of 36 genes, 17 genes are associated with *QTLs* for salt, cold drought, and bacterial stress, and are therefore recommended to design the new stress-resistant cultivars which can provide sustainable yield in stress-specific conditions. Trans-Zeatin (tz) is an active form of cytokinin involved in managing environmental stress, the cytokinin pathway has been widely studied and a huge amount of gene expression data are available in public repositories (Edgar et al., 2002).

## Cytokinin and Linked Molecular Marker for Yield and Yield Related Traits in Wheat

The main pigment in crop plants responsible for photosynthesis is chlorophyll, including chlorophyll a and b, and the key photosynthetic pigment in chloroplasts and its amount directly affects the plant’s photosynthetic efficiency (Araus et al., 1997; Thomas et al., 2005). Its abundance and stability in the leaf significantly affects the grain filling, grain weight, and eventually the actual yield of the crops (Shao et al., 2013). Research findings reveal that cytokinin (*CTK*) can greatly increase leaf chlorophyll content, chloroplast stability, and net photosynthetic rate (Shao et al., 2012; Ding et al., 2013). However, in wheat crop little is known about the association of *Tackx* gene with chlorophyll content and grain weight. The cytokinin can effectively increase chlorophyll content and chloroplast stability, but it is irreversibly inactivated by cytokinin oxidase (*CKX*). Cheng et al. (2015) carried out an experiment with an aim to identify variations of wheat *TKX* (*Tackx*) genes and their association with grain weight and wheat chlorophyll level, and validating the effect of targeted *Tackx* gene on these two traits. Their findings indicated a variation of *Tackx4*, that was proven to be significantly associated with chlorophyll content and grain weight in the RIL populations and also identified two *Tackx4* patterns *viz*., one with two co-segregated fragments (*Tackx4-1/Tackx4-2*) containing 618 bp and 620 bp in size, and another with no PCR product. These two genotypes were designated as genotype-A and genotype-B, respectively. Their mapping analysis reveals that *Tackx4* was closely linked to *Xwmc169* on chromosome 3AL, as well as co-segregated with a major quantitative trait locus (QTL) for both grain weight and chlorophyll content and this *QTL* explained 8.9%–22.3% of the phenotypic variations for the two traits across the four cropping seasons, whereas previous researchers identified the multiple copies of *CKX* genes on chromosome 3DS of wheat, including *Tackx2.1 and Tackx2.2* (Zhang et al., 2011). Previous researchers have also identified the several *QTLs* for yield and yield related traits detected on wheat chromosome 3A which explain 4.1 to 14.27 percent of phenotypic variations in different environments (Kumar et al., 2007; Cuthbert et al., 2008; Wang et al., 2009). For chlorophyll content and photosynthesis, many more *QTLs* have been found on wheat chromosomes 1A, 1D, 2A, 2D, 3B, 4A, 5A, 5B, 5D, 6A, 6D, 7A, and 7D (Sukumaran et al., 2015). Such useful findings may be explored for improving the accuracy and effectiveness of marker assisted selection for chlorophyll level and grain weight in wheat breeding to develop promising genotypes.

## Harnessing the Genetic Resources for Improving the Nutrients, Yield, and Other Desirable Traits

**FIGURE 3:**
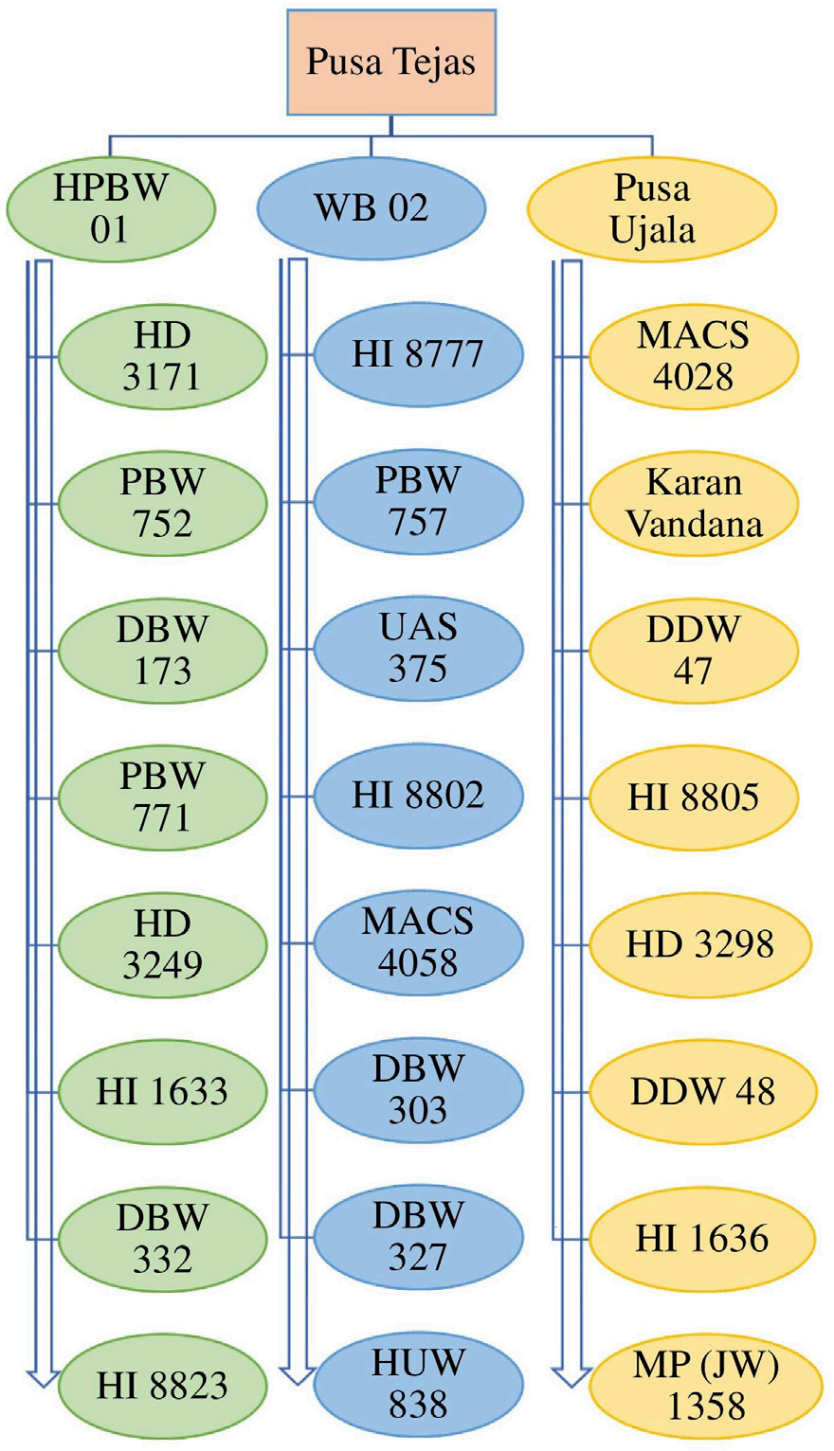
Name of 28 Bio-fortified wheat varieties developed by research institutes in the country.

To continue the challenge, the improved wheat genotypes had satisfactory yield and see improvement in other traits but somehow lacked the necessary nutrients for the human body. In light of this happening in the recent past, about 28 bio-fortified wheat genotypes (Figure 3) have been developed by various agricultural institutes of the country having enough important nutrients and improvement in other desirable traits (Yadava et al., 2022) (Table 1). Besides this, many other studies have been carried out regarding the transport of micronutrients in crops and to enhance their content, such as Wang et al. (2014), Qin et al. (2016), Connorton et al. (2017), Singh et al. (2017), Beasley et al. (2019) and Liang et al. (2019). Hence, in order to improve wheat genotypes for nutrients and other desirable traits including resistance for biotic and abiotic stresses, these genotypes may be chosen as donor parents in crossing/breeding programmes. Besides this, published studies report that some genotypes have a unique genetic make-up that means if a foliar spray of chemical fertilizers is provided, then they are synthesizing enough nutrients into grains thus, genotypes could be explored through smart breeding at a molecular level to understand genetics of such targeted traits with aim to develop superior genotypes. Similarly, association of morpho-physiological traits with resistance to spot blotch in wheat such as leaf angle (Joshi and Chand 2002; Prasad et al., 2013), leaf tip necrosis (Joshi et al., 2004a), and stay green trait (Joshi et al., 2007a) have been studied and recommended to explore breeding of the promising genotypes as these phenotypic traits are strongly associated with resistance to spot blotch disease. Stay-green is a key trait of wheat can not only enhance the yield of wheat crops because of its efficiency in photosynthesis but is also able to contribute for resistance to heat, spot blotch and other stresses. This unique trait can also be used as a morphological marker for selecting the spot blotch disease resistance wheat among the large populations and explore the breeding of promising genotypes through hybridization. Cytokinins are well known as the most potent general coordinator between the stay-green trait and senescence of plant species. Schwarz et al. (2020) reported that expression of cytokinins can directly increase seed yield, grain numbers, and seed size of the concerned crops, and it has a significant response against environmental stressors as well (Cortleven et al., 2019). The examinations of endogenous cytokinin levels under various conditions reveals that cytokinin metabolism is highly regulated during the response to abiotic stress (Zwack and Rashotte, 2015). Because of their recognized effects on increasing seed number and seed size, and effect under stressed conditions and synthesizing important nutrients, the cytokinins may well be the hormone that underpins the second “Green Revolution” as highlighted by Lynch (2007). Virk et al. (2021), have summarized the targeted breeding for micronutrients contents is/was conceived by HarvestPlus which was the challenging international program of CGIAR. This is a biofortified breeding program including high-throughput micronutrient phenotyping, genomic selection coupled with speed breeding for accelerating genetic gains with an aim to develop the biofortified genotypes through biofortification for three important micronutrients, namely iron (Fe), zinc (Zn), and provitamin A (PVA), needed for human health as they have gained momentum in the 21st century. The plan of Harvest Plus, along with its global consortium partners, is to significantly increase the quantity of Fe, Zn, and PVA in staple crops as well as release bio-fortified wheat varieties with huge potential across the globe. Such bio-fortified genotypes could be used as parent/donors in future breeding programs for crop improvement. The key center of CGIAR *viz*., CIMMYT has an outstanding role to develop superior wheat genotypes which have enough zinc and other required nutrients (Yuan et al., 2019; Jigly et al., 2019).

**TABLE 1:** List of 28 bio-fortified wheat varieties released in country better for nutrients quality parameters.

Variety	Descriptions
WB 02	WB 02 wheat variety is developed by ICAR-Indian Institute of Wheat & Barley Research, Karnal which is released in 2017. It is rich in iron (40.0 ppm) and zinc (42.0 ppm) in comparison to 28.0–32.0 ppm iron and 30.0–32.0 ppm zinc in popular varieties. The grain yield of this variety is 51.6 q/ha, matured in142 days, suitable for irrigated timely sown conditions in *rabi* and recommended for cultivation in Punjab, Haryana, Delhi, Rajasthan, Western Uttar Pradesh and oter states
HPBW 01	HPBW 1 wheat variety is developed by Punjab Agricultural University, Ludhiana under ICAR-All India Coordinated Research Project on Wheat & Barley which is released in 2017. It is rich in iron (40.0 ppm) and zinc (40.6 ppm) in comparison to 28.0–32.0 ppm iron and 30.0–32.0 ppm zinc in popular varieties. This variety yielded 51.7 q/ha, matured in 141 days, suitable for irrigated timely sown conditions and recommended for cultivation in Punjab, Haryana, Delhi, Rajasthan and other states of the country
Pusa Tejas (HI 8759)	Pusa Tejas wheat variety is also known as HI 8759 durum wheat, developed by ICAR-Indian Agricultural Research Institute, Regional Station, Indore and released in 2017. It is rich in protein (12.0%), iron (41.1 ppm) and zinc (42.8 ppm) in comparison to 8%–10% protein, 28.0–32.0 ppm iron and 30.0–32.0 ppm zinc in popular varieties. Its yield is 57.0 q/ha, matured is 117 days, suitable for irrigated timely sown conditions in *rabi* and recommended for its cultivation in Madhya Pradesh, Chhattisgarh, Gujarat, Rajasthan and Uttar Pradesh
Pusa Ujala (HI 1605)	Pusa Ujala wheat variety is developed by ICAR-Indian Agricultural Research Institute, Regional Station, Indore which is released in 2017. It is rich in protein (13.0%) and iron (43.0 ppm) in comparison to 8%–10% protein and 28.0–32.0 ppm iron in popular varieties, having grain yield 30.0 q/ha, maturity time 105 days, suitable for timely sown restricted irrigated conditions in rabi season and recommended for cultivation in Maharashtra and Karnataka
HD 3171	HD 3171 wheat variety is developed by ICAR-Indian Agricultural Research Institute, New Delhi and released in 2017. This is rich in zinc (47.1 ppm) in comparison to 30.0–32.0 ppm in popular varieties. The grain yield of this variety is 28.0 q/ha, maturity time is 122 days, suitable for timely sown rainfed conditions in rabi and recommended for cultivation in Eastern Uttar Pradesh, Bihar, Jharkhand, Odisha, West Bengal, Assam and plains of North Eastern States
HI 8777	HI 8777 is durum wheat developed by ICAR-Indian Agricultural Research Institute, Regional Station, Indore and released in 2018. It is rich in iron (48.7 ppm) and zinc (43.6 ppm) in comparison to 28.0–32.0 ppm iron and 30.0–32.0 ppm zinc in popular varieties. The grain yield of this variety is 18.5 q/ha, maturated in 108 days, suitable for timely sown rain-fed conditions in *rabi* season and recommended for its cultivation in Maharashtra, Karnataka and plains of Tamil Nadu
MACS 4028	MACS 4028 is a durum wheat developed by Agharkar Research Institute, Pune under ICAR-All India Coordinated Research Project on Wheat & Barley and released in 2018. It is rich in protein (14.7%), iron (46.1 ppm) and zinc (40.3 ppm) in comparison to 8%–10% protein, 28.0–32.0 ppm iron and 30.0–32.0 ppm zinc in popular varieties. It gives grain yield 19.3 q/ha, maturated in 102 days, suitable for rainfed, low fertility, timely sown conditions in *rabi* and recommended for cultivation in Maharashtra and Karnataka
PBW 752	PBW 752 wheat variety is developed by Punjab Agricultural University, Ludhiana under ICAR-All India Coordinated Research Project on Wheat & Barley and released in 2018. It is rich in protein (12.4%) in comparison to 8–10% in popular varieties, having grain yield 49.7 q/ha, matured in 120 days, suitable for late sown irrigated conditions in *rabi* season and recommended to cultivate for Punjab, Haryana, Delhi, Rajasthan and other states
PBW 757	It is developed by Punjab Agricultural University, Ludhiana under ICAR-All India Coordinated Research Project on Wheat & Barley and released in 2018. Contains high zinc (42.3 ppm) in comparison to 30.0–32.0 ppm zinc in popular varieties. Yield of this variety is 36.7 q/ha, maturity time is 104 days, suitable for very late sown irrigated conditions in *rabi* season and recommended for cultivation in Punjab, Haryana, Delhi, Rajasthan and other states
Karan Vandana (DBW 187)	Karan Vandana wheat variety is developed by ICAR-Indian Institute of Wheat & Barley Research, Karnal and released in 2018 and 2020. It is rich in iron (43.1 ppm) in comparison to 28.0–32.0 ppm in popular varieties, having grain yield 48.8 q/ha in North Eastern Plains Zone (NEPZ), 61.3 q/ha in North Western Plains Zone (NWPZ) and 75.5 q/ha in high fertility conditions. Variety in matured in 120 days (NEPZ), 146 days (NWPZ) and 158 days (Highfertility) conditions. Suitable for timely sown irrigated and fertlity conditions in *rabi* season and recommended for cultivation in Punjab, Haryana, Delhi, Rajasthan and other states
DBW 173	DBW 173 is developed by ICAR-Indian Institute of Wheat & Barley Research, Karnal released in 2018. It is rich in protein (12.5%) and iron (40.7 ppm) in comparison to 8%–10% protein and 28.0–32.0 ppm iron in popular varieties, having grain yield 47.2 q/ha, matured in 122 days, suitable for late sown irrigated conditions in rabi season and recommended for cultivation in Punjab, Haryana, Delhi, Rajasthan and other states
UAS 375	This wheat variety is developed by University of Agricultural Sciences, Dharwad under ICAR-All India Coordinated Research Project on Wheat & Barley, released in 2018, rich in protein (13.8%) in comparison to 8%–10% in popular varieties. Produces 21.4 q/ha grain yield, matured in 103 days, suitable for timely sown rainfed conditions in rabi season and recommended for cultivation in Maharashtra and Karnataka
DDW 47	DDW 47 is developed by ICAR-Indian Institute of Wheat & Barley Research, Karnal, released in 2020P. Variety rich in protein (12.7%) and iron (40.1 ppm) in comparison to 8%–10% protein and 28.0–32.0 ppm iron in popular varieties, having grain yield 37.3 q/ha, maturity time 121 days, suitable for timely sown restricted irrigated conditions in rabi season and recommended for cultivation in Madhya Pradesh, Gujarat, Rajasthan and Chhattisgarh
PBW 771	PBW 771 variety is developed by Punjab Agricultural University, Ludhiana under ICAR-All Indian Coordinated Research Project on Wheat & Barley and released in 2020. It is rich in zinc (41.4 ppm) in comparison to 30.0–32.0 ppm in popular varieties. It has 50.3 q/ha grain yield, matured in 120 days, suitable for late sown irrigated conditions in *rabi* season and recommended for cultivation in Punjab, Haryana, Delhi, Rajasthan and other states
HI 8802	It is durum wheat developed by ICAR-Indian Agricultural Research Institute, Regional Station, Indore and released in 2020. It is rich in protein (13.0%) in comparison to 8%–10% in popular varieties, having grain yield: 29.1 q/ha, matured in 109 days, suitable for timely sown in rain-fed and recommended for cultivation in Maharashtra, Karnataka and plains of Tamil Nadu
HI 8805	HI 8,805 (durum wheat) is developed by ICAR-Indian Agricultural Research Institute, Regional Station, Indore and released in 2020. It is rich in protein (12.8%) and iron (40.4 ppm) in comparison to 8%–10% protein and 28.0–32.0 ppm iron in popular varieties. It has grain yield 30.4 q/ha, matured in 105 days, suitable for timely sown in rainfed conditions and recommended for its cultivation in Maharashtra, Karnataka and plains of Tamil Nadu
HD 3249	HD 3,249 variety is developed by ICAR-Indian Agricultural Research Institute, New Delhi and released in 2020. It is rich in iron (42.5 ppm) in comparison to 28.0–32.0 ppm in popular varieties. It has yielded 48.8 q/ha, matured in 122 days, suitable for timely sown irrigated conditions in rabi season and recommended for cultivation in Eastern Uttar Pradesh, Bihar, Jharkhand, West Bengal (excluding Hills), Odisha, Assam and plains of North Eastern States
MACS 4058	MACS 4,058, is durum wheat which is developed by Agharkar Research Institute, Pune under ICAR-All India Coordinated Research Project on Wheat & Barley and released in 2020. It is rich in protein (14.7%), iron (39.5 ppm) and zinc (37.8 ppm) in comparison to 8%–10% protein, 28.0–32.0 ppm iron and 30.0–32.0 ppm zinc in popular varieties, it has 29.6 q/ha grain yield, matured in 102 days, suitable for timely sown restricted irrigated conditions in *rabi* season and recommended for cultivation in Maharashtra and Karnataka
HD 3298	HD 3,298 is developed by ICAR-Indian Agricultural Research Institute, New Delhi which is of released in 2020. It is rich in protein (12.1%) and iron (43.1 ppm) in comparison to 8%–10% protein and 28.0–32.0 ppm iron in popular varieties, having grain yield 43.7 q/ha, matured in 103 days, suitable for very late sown n irrigated conditions and recommended for cultivation in Punjab, Haryana, Delhi, Rajasthan and other states
HI 1633	This is developed by ICAR-Indian Agricultural Research Institute, Regional Station, Indore and released in 2020. It is rich in protein (12.4%), iron (41.6 ppm) and zinc (41.1 ppm) in comparison to 8%–10% protein, 28.0–32.0 ppm iron and 30.0–32.0 ppm zinc in popular varieties. Variety has 41.7 q/ha grain yield, matured in 100 days, suitable for late sown irrigated conditions and recommended for cultivation in Maharashtra, Karnataka and plains of Tamil Nadu
DBW 303	DBW 303 is developed by ICAR-Indian Institute of Wheat & Barley Research, Karnal and released in 2020. It is rich in protein (12.1%) in comparison to 8%–10% protein in popular varieties. Variety produces 81.2 q/ha grain yield, matured in 156 days, suitable for irrigated early sown and high fertility conditions in rabi and recommended for its cultivation in Punjab, Haryana, Delhi, Rajasthan and other states
DDW 48	DDW 48 is durum wheat, developed by ICAR-Indian Institute of Wheat & Barley Research, Karnal and released in 2020. It is rich in protein (12.1%) in comparison to 8%–10% protein in popular varieties, having grain yield 47.4 q/ha, matured in 111 days, suitable for timely sown irrigated conditions in rabi season and recommended for cultivation in Maharashtra, Karnataka and plains of Tamil Nadu
DBW 332	Variety is developed by ICAR-Indian Institute of Wheat & Barley Research, Karnal which released in 2021. This variety is rich in protein (12.2%) and zinc (40.6 ppm) in comparison to popular variety. Its yield capacity is 78.3 q/ha, maturity time is 156 days and suitable for early sown irrigated conditions in *rabi* season for different states of the country
DBW 327	This variety is developed by ICAR-Indian Institute of Wheat & Barley Research, Karnal and released in 2021. Its contains high zinc (40.6 ppm), yielded 79.4 q/ha, matured in 155 days and suitable for its cultivation early sown irrigated conditions in Punjab, Haryana, Delhi, Rajasthan, Western Uttar Pradesh, etc.
HI 1636	It is developed by ICAR-Indian Agricultural Research Institute, Regional Station, Indore and released in 2021. Variety contains high zinc (40.4 ppm), has 56.6 q/ha grain yield, matured in 119 days and suitable for timely sown irrigated conditions in *rabi* for Madhya Pradesh, Chhattisgarh, Gujarat, Rajasthan, etc.
HI 8823	HI 8823 wheat variety is developed by ICAR-Indian Agricultural Research Institute, Regional Station, Indore and released in 2021. Rich in protein (12.1%) and zinc (40.1 ppm) in comparison to popular varieties. It gives 38.5 q/ha grain yield, maturity in 122 days and suitable for timely sown irrigated conditions in *rabi* for the states like Madhya Pradesh, Chhattisgarh, Gujarat, Rajasthan, etc.
HUW 838	HUW 838 wheat variety is developed by Banaras Hindu University, Varanasi under ICAR-All India Coordinated Research Project on Wheat & Barley and released in 2021. It contains high zinc (41.8 ppm), yielded 51.3 q/ha grain yield, matured in 148 days and suitable for early sown irrigated conditions in *rabi* for the states such as Punjab, Haryana, Delhi, Rajasthan, Western Uttar Pradesh, etc.
MP (JW) 1,358	This wheat variety is developed by Jawahar Lal Nehru Krishi Viswavidhyalaya, Zonal Agricultural Research Station, Powarkheda under ICAR-All India Coordinated Research Project on Wheat & Barley and released in 2021. It is rich in protein (12.1%) and iron (40.6 ppm) in comparison to popular wheat varieties. Its grain yield is 56.1 q/ha, maturity time is 105 days and suitable for early sown irrigated conditions in for Maharashtra, Karnataka and plains of Tamil Nadu.

## Important Approches/Technology Utilised for Crop Bio-Fortification

**FIGURE 4:**
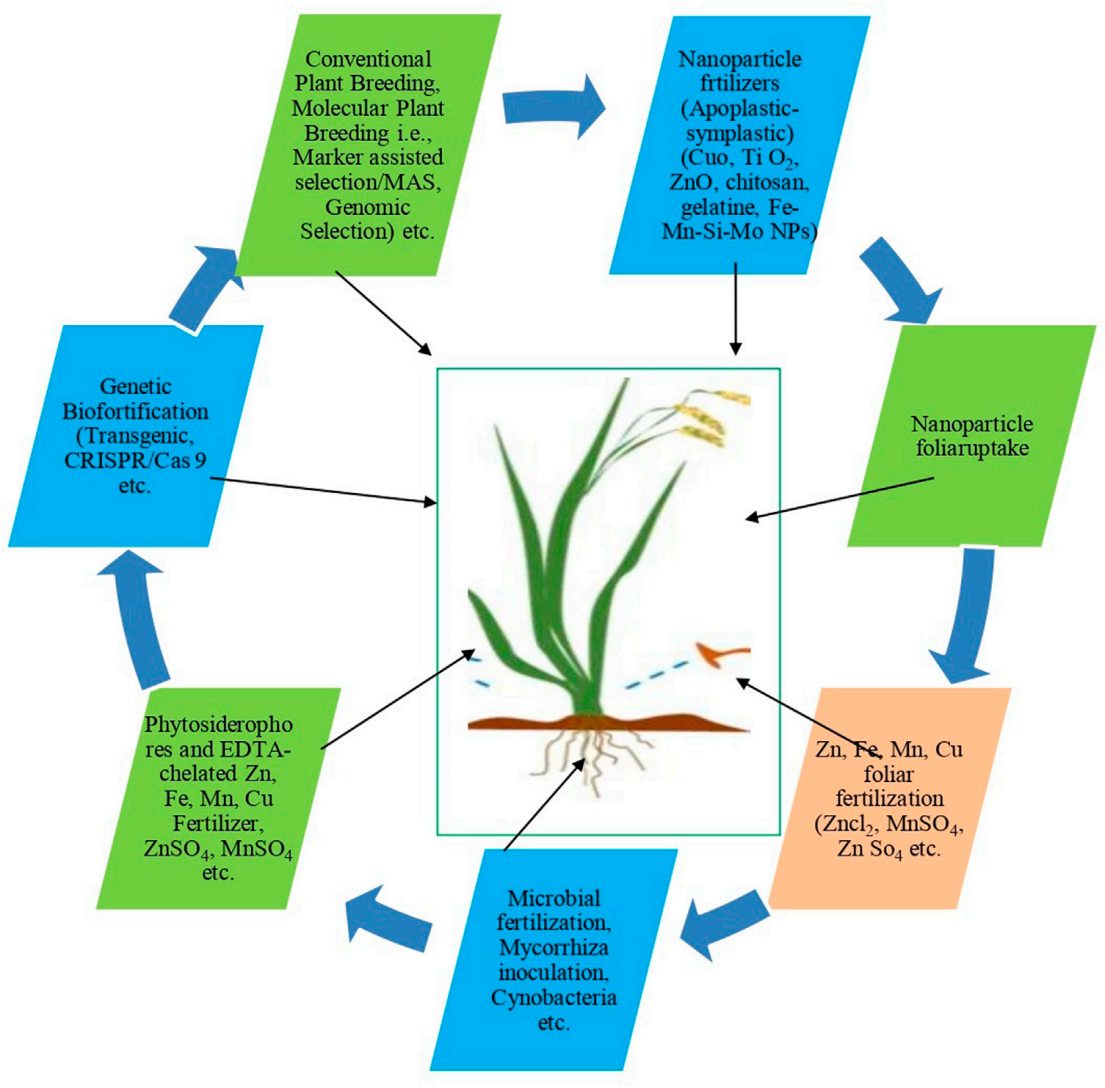
Important approches/technology uses for crop bio-fortification.

Reports indicate that more than 820 million people in the world are hungry and two billion people are suffering from micronutrient deficiencies (Kane et al., 2015; Boliko et al., 2019; UNEP. 2021). Most of the crop plants can accumulate micronutrients however; some main plants lack the adequate amounts of such nutrients *viz*., Fe and Zn in the edible parts (Wakeel et al., 2018), for instance, basic/staple crops such as rice, wheat, and maize contain low amounts of Zn and Fe (Shariatipour and Heidari 2020). In recent research, it is strongly stated that micronutrient deficiencies increase susceptibility to many infectious diseases, including Covid-19 (Akhtar et al., 2021). Therefore, more attention has been made to enhance such import nutrients in the crop plants through bio-fortification, which is an effective strategy to combat micronutrient deficiency. Different useful approaches of bio-fortification are being used to improve the nutritional value of plants, to overcome nutritional the problems presented in Figure 4, and also described by Koç and Karayiğit (2022), with a remark that bio-fortification is a cost-effective and sustainable agricultural strategy for increasing the bioavailability of essential elements/nutrients in the edible parts of plants and reducing malnutrition. Further, they have also pointed out that genetic bio-fortification based on genetic engineering such as increasing or manipulating the expression of genes that affect the regulation of metal homeostasis and carrier proteins that serve to increase the micronutrient contents and greater productivity through *CRISPR-Cas9* (bacterial Clustered Regularly Interspaced Short Palindromic Repeats) technology can be considered as a promising high-potential strategy or modern and very advanced GM technology for solving the micronutrient deficiency problem and this technique was reported for the first time by Jinek et al., 2012. By modifying the germ line cells, *Crispr-cas* technology has the potential to develop transgenics without involving transformation and tissue culture plants (Malik and Maqbool, 2020). Eckerstorfer et al. (2019) stated that, across the globe, new genetic modification technique (*nGMs*) approaches, particularly genome editing, are used in basic and applied research. In parallel to classic genetically modified technology a wide range of *nGM* techniques are being developed for the (genetic) modification of organisms, including plants, for research purposes or for the development of crops for agricultural purposes. These *nGMs* are also referred to as “new techniques” or “new breeding techniques” for improving targeted traits (Lusser et al., 2012; SAM, 2017).

## Role of Cytokinins Under a-Biotic Stress Response

Besides the important role of cytokinins for plant growth and development in normal conditions, they play a very crucial role for crop plants under abiotic stressed conditions such as heat stress, drought stress, cold stress, and salt stress (Liu et al., 2020) which is summarized as follows

It is known that high temperatures (heat/terminal heat) can reduce chlorophyll content and photochemical efficiency of plant leaves, resulting in negative impact on the photosynthetic process (Zhang et al., 2017). Higher temperature also increases the production of reactive oxygen species (ROS) and protease activity, leading to leaf senescence (Hu et al., 2020). It increases the production of ROS, and elevated cytokinins can stimulate the antioxidant system to remove ROS (Xu et al., 2009). According to Skalak et al. (2016), hormonal analysis of the proteome and transcriptome also confirms that cytokinins play an important role in plant resistance to heat stress, and most of the heat shock (HS) proteins which are upregulated by increasing the cytokinins. Since accumulation of endogenous cytokinins can maintain normal plant growth under high temperature, heat stress tolerance of plants can be improved by increasing the content of endogenous cytokinins. Insertion of *isopentenyl transferase* (*IPT*) in *Arabidopsis* at the seedling stage which significantly improves the level of endogenous cytokinin and thus enhances the tolerance in high temperatures (Skalak et al., 2016). Drought is one of the major factors which can inhibit the plant physiological functions, including reduction of photosynthesis, crop yield, and accelerated senescence (Liu et al., 2012). The possibility of improving the drought tolerance of plants by regulating cytokinin levels depends on stress duration, soil water potential, and plant dehydration rate (Veselov et al., 2017). In response to drought, up and downregulation of endogenous cytokinins can enhance the degree of drought tolerance (Werner et al., 2010). Some findings reveal that during drought stress, the accumulation of plant endogenous cytokinins is reduced, and this reduction can enhance the plant drought tolerance *via* various physiological responses including stomatal closure (Naidoo and Naidoo 2018). Cytokinin is downregulated, leading to the expansion of the root system and a high root to shoot ratio, which increases the water absorption area of roots. Relatively small shoot and leaf area as compared to roots can effectively decrease transpiration rate (Lubovska et al., 2014) and therefore, the whole plant can maintain high relative water content and improve drought tolerance. Cold (low temperature) is another stress which can affect the plant cells by hardening the membrane system and interfering with all membrane-related processes (Liu et al., 2016), low temperatures can also lead to the accumulation of ROS, due to the decrease of antioxidant enzyme activity making the ROS scavenging system unable to work normally, and in turn, the excessive accumulation of ROS will have harmful effects on the membrane, resulting in leakage of ions and cell metabolism disorder (Sui, 2015). Like other stress, salt stress can hamper the physiological and biochemical processes of crop plants. Sodium-ion (Na+) accumulation in plants can lead to the disorder of ion homeostasis, the imbalance of potassium ion (K+)/Na + ratio, and Na + ion toxicity (Liu et al., 2017) and cause oxidative stress, which damages the cell membrane, causes ion leakage, or direct damage to proteins and other macromolecules, leading to cytotoxicity and even cell death (Lin et al., 2018). Avalbaev et al. (2016) reported that pretreatment of wheat with exogenous methyl jasmonate (*MeJA*) can maintain the high content of cytokinin by decreasing the *CKX* transcription level induced by salt stress, and enhance salt tolerance level. Therefore, by spraying the exogenous cytokinins onto plants, the salt tolerance property can be enhanced.

## Conclusion

Wheat is considered one of the most economically important cereal crops in the world. Its productivity is high, but increasing consumption and changing climate indicates the need for further improvement in its yield potential. Climate change is expected to continue posing biotic/abiotic stresses, and if current trends continue, many parts of the planet will become hostile to agriculture. On the other hand, because of the rapidly growing population and the changing climate, demand for wheat is expected to grow faster than the other major crops. Therefore, investments in exploring and harnessing existing genetic resources including bio-fortified wheat and information regarding the role of cytokinins under normal and adverse conditions for increasing the yield, grains containing enough nutrients and having a sufficient defense response, impact of morphological markers *viz*., stay green traits, leaf tip necrosis, leaf angle molecular markers, and other useful genetic information in order to produce enough food grains that are also rich in the required nutrients to ensure food security in the 21st century is the need of the day.

Since cytokinins are the most important endogenous substances moderating the physiological and molecular responses, thay have a key role during completion of the life cycle of plants in order to give a satisfactory yield. So, plant breeders could directly target the cytokinins to improve targeted traits by utilizing minimum input, as cytokinins are known to be a key driver of seed yield and it may well be the hormone that underpins the second green revolution. The Green Revolution boosted crop yields during the mid 20th century by introducing dwarf genotypes of wheat capable of responding to a higher dose of fertilization and enough irrigation without lodging. Now there is need of a second Green Revolution to meet out the demand of a rapidly growing population. The Green Revolution was based on crops responsive to high soil fertility however, now there is need to develop the genotypes of wheat crops which can perform better under low input, low soil fertility, under stressed conditions including heat, terminal heat, drought, metal toxicity, and under biotic stresses as well. By keeping the above facts in mind, exploring genetic resources, harnessing the cytokinin key hormones, and applying updated molecular breeding approaches, plant breeders can develop the superior and stable genotypes which will be able to cater to the food demand of the needy population.
